# Perceptions of persistent idiopathic facial pain: a comprehensive study of adults in Ha’il city, Saudi Arabia

**DOI:** 10.22514/jofph.2025.033

**Published:** 2025-06-12

**Authors:** Abdullah F. Alshammari, Ahmed A. Madfa, Amal R. Alrashidi, Ebtisam A. Alshdokhy, Sattam S. Alshammari, Khlood A. Alkurdi

**Affiliations:** ^1^Department of Basic Dental and Medical Science, College of Dentistry, University of Ha’il, 55473 Ha’il, Kingdom of Saudi Arabia; ^2^Department of Restorative Dental Science, College of Dentistry, University of Ha’il, 55473 Ha’il, Kingdom of Saudi Arabia; ^3^Dental Research Centre, College of Dentistry, University of Ha’il, 55473 Ha’il, Kingdom of Saudi Arabia; ^4^Department of Preventive Dental Science, College of Dentistry, University of Ha’il, 55473 Ha’il, Kingdom of Saudi Arabia; ^5^Ministry of Health, Qassim Health Cluster, King Saud Hospital, 51911 Unayzah, Kingdom of Saudi Arabia

**Keywords:** Idiopathic facial pain, Perception, Pharmacotherapy, Behavioural therapy, Treatment, Saudi Arabia

## Abstract

**Background**: Persistent idiopathic facial pain (PIFP) is a complex condition characterized by chronic, unexplained facial pain that significantly impacts patients’ quality of life and remains poorly understood by the general public. This research aimed to assess the knowledge and understanding of PIFP among the general population of Ha’il, Saudi Arabia. **Methods**: This cross-sectional study examined Ha’il residents’ attitudes and levels of knowledge regarding PIFP and investigated the associated factors. Anonymised surveys were distributed to 350 respondents between November 2023 and March 2024. The original survey draft was based on a combination of previously published research, the Facial Pain Association, and previously validated questionnaires addressing similar objectives. A standardised survey scheme was designed, pre-coded and validated. The refined survey instrument was then transformed into an online questionnaire using Google Survey© 2023 and distributed. **Results**: In total, 254 respondents filled out the survey. The chi-square test was utilised to assess knowledge and attitudes in relation to participants’ sociodemographic characteristics. Spearman’s rank correlation coefficient (*p* < 0.05), odds ratios and confidence intervals were calculated to assess the relationship between attitudes and knowledge. Binary logistic regression analysis was performed to identify predictors of high knowledge levels and positive attitudes. The participants showed moderate knowledge of PIFP, with 51.24% correct responses. Notably, 96.90% identified dental issues, infections and nerve abnormalities as key factors. Gender influenced perceptions, with 30.9% of women and 45.9% of men downplaying PIFP’s significance, while age, education and occupation had minimal impact (*p* > 0.05). **Conclusions**: This study emphasises the critical need for targeted educational programs to address misconceptions and information gaps around PIFP. The information gained highlights the need for an advanced approach to health education and communication that is tailored to the unique cultural and demographic characteristics of Ha’il, Saudi Arabia.

## 1. Introduction

Persistent idiopathic facial pain (PIFP), formerly recognised as atypical facial pain (AFP), is a medical disorder characterised by continuous, pulsating pain in the teeth or face with no anatomical link. As per the 2018 International Classification of Headache Disorders (ICHD) third edition, PIFP is defined as chronic facial and/or oral pain, presenting in various forms but occurring daily for over 2 hours per day and persisting for more than 3 months, without any detectable neurological abnormalities [1, 2, 3, 4]. In Saudi Arabia, sociocultural perceptions of pain and healthcare-seeking behaviour may differ significantly from those in Western populations, on which most PIFP studies have been conducted. Thus, exploring PIFP in Ha’il, Saudi Arabia, provides an opportunity to study unique influences, potentially unveiling new insights into the condition’s epidemiology and management [5].

PIFP affects approximately 1% of people experiencing orofacial pain, which comprises various forms of pain in the head and neck caused by a set of conditions. Although less prevalent, PIFP still occurs from time to time in the overall population, particularly among women and people with an average age of 40 years. Differences in published data exist regarding the frequency of PIFP in the general population. A population-based study in Germany estimated the prevalence of PIFP at approximately 0.03%, while a recent study in Turkey reported a higher prevalence of 0.202% (202 per 100,000 individuals) [6, 7]. PIFP generally appears as a gentle and faint ache that does not align with a peripheral nerve course. Pain typically starts at the nasolabial fold or chin and can spread to the jaw, face and neck. It usually begins on one side but can transition to both sides. Triggers may involve small surgeries or injuries, and the condition can also arise suddenly [4, 8].

Precise differential diagnosis is crucial to avoid treatment delays, especially when dealing with complex conditions such as trigeminal neuralgias and primary headaches [9]. PIFP is identified through clinical and neurological assessments following the exclusion of dental factors [10]. Imaging techniques such as Computed Tomography (CT) scans, Magnetic Resonance Imaging (MRIs) and facial X-rays play a vital role in ruling out major abnormalities [11]. The diagnosis of PIFP is typically considered a final option or a way to rule out other conditions and is usually made only after other therapies have been unsuccessful [12]. PIFP can significantly affect quality of life, causing chronic pain that disrupts daily activities and sleep [8]. According to the literature, PIFP is associated with psychological disorders, with studies indicating a high prevalence of anxiety and depression [1].

Management strategies include pharmacotherapy and behavioral therapy, with evidence supporting pain relief through hypnosis sessions, although additional psychological support is often required [13]. Medications such as tricyclic antidepressants (25 to 100 mg/day), serotonin-noradrenaline reuptake inhibitors (*e.g.*, venlafaxine) and selective serotonin reuptake inhibitors (*e.g.*, fluoxetine) are all effective [3]. In cases where medications are ineffective, pulsed radiofrequency (PRF) therapy of the pterygopalatine ganglion has demonstrated significant results with minimal side effects, although temporary bradycardia has been observed in standard PRF therapy [14]. Botulinum toxin injections have also been explored as recent advancements in PIFP treatment. On the other hand, surgical interventions, including deep-brain stimulation and trigeminal artery decompression, have largely been unsuccessful in treating PIFP [15, 16, 17, 18, 19].

Dentists may come across patients suffering from PIFP during treatment. The cause of PIFP is currently unknown, making treatment challenging and often ineffective. PIFP is also difficult to diagnose because its symptoms overlap with those of other orofacial syndromes. Therefore, the process of exclusion, known as differential diagnosis, is crucial when creating a successful treatment plan to prevent unnecessary, invasive procedures. Poorly handling PIFP has serious physical, psychological and social effects and causes significant financial losses due to healthcare expenses and decreased productivity. Understanding and effectively treating PIFP is not just a clinical challenge but a societal imperative in medicine and dentistry and for the national healthcare system. Literature about PIFP in Saudi Arabia is scarce. To the best of our knowledge, no study has yet investigated the level of awareness and understanding regarding PIFP in the Saudi Arabian population. Hence, the present study aimed to assess the level of awareness and understanding of the general population regarding PIFP in the city of Ha’il, Saudi Arabia. It also focused on attitudes towards PIFP.

## 2. Materials and methods

### 2.1 Study design

This cross-sectional study was conducted between November 2023 and March 2024. It targeted the knowledge and attitudes of individuals living in Ha’il city towards PIFP. The study included adults aged 18 years and older who had resided in Ha’il, Saudi Arabia, for at least 6 months and could read and write in Arabic or English. Participants were required to have access to an internet-enabled device and to provide informed consent. Exclusion criteria included incomplete survey responses, recent residency in Ha’il (less than 6 months) and involvement in the design, administration or analysis of the study to avoid potential conflicts of interest.

### 2.2 Sample size

The Raosoft® sample size calculator was used to calculate the study’s sample size. The suggested sample size was 236, with a 50% response distribution, a 95% confidence level and a 5% acceptable margin of error. In total, 350 responses were targeted to minimise errors.

### 2.3 Survey construction and dissemination

A standardised online survey scheme was structured, pre-coded and validated, with the initial survey draft created based on previously reviewed and published literature [20], the Facial Pain Association (FPA) [21] and validated questionnaires that previously addressed our objectives [22]. It focused on three major topics: (1) participants’ perception of PIFP, defined as their awareness and understanding of its causes, symptoms and impact on quality of life; (2) participants’ assertiveness towards PIFP, referring to their attitudes and proactive behaviours, such as recognising its seriousness and seeking professional help; and (3) identification of potential correlations between various factors, such as participant demographics, educational background and work experience, and their knowledge and attitudes regarding PIFP.

The researchers participated in calibration training prior to the evaluation. Before beginning data collection, the researchers received training based on previously defined standards and deviations. After reviewing the literature, the questionnaires for this study were created. The questionnaire’s reliability was confirmed through a pilot test with 15 participants. College members from the Department of Basic Dental and Medical Science at the University of Ha’il, Saudi Arabia, collaborated with experts to confirm the questionnaire’s relevance to the survey’s topic. The questionnaire was validated in two steps. It was first examined by a focus group consisting of experienced oral medicine specialists and a facial pain expert to consider the relevance, convenience and importance of the questions. Then, a peer review was carried out, wherein senior dental staff members were invited to evaluate and provide feedback on the survey tool, streamline and condense the data collection tool, clarify any unclear questions and eliminate any repetitive elements. Their suggestions were integrated into the final version before the survey was distributed. The final edition had three sections and 28 closed-ended multiple-choice questions. Nine questions in the first portion were used to ascertain the demographics and professional standing of the respondents. The second segment comprised seven questions that assessed the respondent’s perceived level of PIFP knowledge. The third segment of the survey consisted of seven items about respondents’ attitudes towards PIFP.

The refined survey instrument was then transformed into an online questionnaire using Google Survey© 2023, as it is user-friendly and reachable on multiple online browsers. The survey was validated by piloting it on 15 participants prior to dissemination to ensure its practicability and make necessary amendments. This was done electronically between August 2023 and September 2023. Social media platforms were utilised to promote the published survey to obtain the desired sample number (the survey questions are presented in Tables 1,2,3). Both Arabic and English versions of the questionnaire were developed as the city of Ha’il harbours multinational communities. Both versions were pre-tested to make sure the original meaning was retained.

**Table 1.** Sociodemographic characteristics of participants (N = 262).

Characteristics		Frequency
1. Are you a resident of Ha’il, Saudi Arabia?		
	Yes	(257) 98.1%
	No	(5) 1.9%
2. Are you 18 years old or above?		
	Yes	(259) 98.9%
	No	(3) 1.1%
3. What is your gender?		
	Male	(91) 35.2%
	Female	(163) 64.8%
4. What is your nationality?		
	Saudi	(237) 95.1%
	Non-Saudi	(17) 4.9%
5. Age (yr)		
	18–28	(100) 39.37%
	29–38	(68) 26.77%
	39–48	(47) 18.50%
	49–58	(22) 8.67%
	59–69	(17) 6.69%
6. What is your level of education?		
	Doctorate	(8) 3.15%
	Master	(16) 6.30%
	Bachelor	(106) 41.73%
	Diploma	(4) 1.57%
	High school	(71) 27.96%
	Middle school	(22) 8.66%
	Primary school	(15) 5.91%
	No education	(12) 4.72%
7. What is your occupation sector?		
	Healthcare sector	(44) 17.32%
	Educational sector	(55) 21.55%
	Retired	(20) 7.67%
	Student	(71) 27.94%
	Military sector	(9) 3.34%
	Governmental sector	(15) 5.80%
	Engineering sector	(2) 0.78%
	Unemployed	(25) 9.83%
	Business	(8) 3.15%
	Administrative	(1) 1.05%
	Private sector	(4) 1.57%
8. What is your monthly income?		
	<3000 SAR	(92) 36.23%
	3000–6000 SAR	(47) 18.50%
	6000–9000 SAR	(46) 18.11%
	9000–15,000 SAR	(45) 17.71%
	>15,000 SAR	(24) 9.55%
9. What is your marital status?		
	Married	(113) 44.49%
	Unmarried	(85) 33.46%
	Divorced	(35) 13.78%
	Widow	(11) 4.33%
	Prefer not to say	(10) 3.94%

*SAR: Saudi Arabian Riyals.*

**Table 2.** Respondents’ level of knowledge about PIFP.

Questions on knowledge about PIFP Total level of participants’ knowledge was 51.24%			
Question	Response	Correct/Incorrect	Frequency
1. The most common causes of PIFP are dental problems, infections, and nerve disorders.			
	True	Correct	(246) 96.90%
	Uncertain	Incorrect	(8) 3.14%
	False		
2. The female gender suffers more from PIFP.			
	True	Correct	(117) 46.06%
	Uncertain	Incorrect	(137) 53.93%
	False		
3. The age range of individuals with PIFP is above 70 years.			
	True	Incorrect	(177) 69.69%
	Uncertain		
	False	Correct	(77) 30.31%
4. PIFP occurs more often in less developed countries.			
	True	Incorrect	(142) 55.90%
	Uncertain	Correct	(112) 44.90%
	False		
5. Antihistamine medication does not treat PIFP.			
	True	Correct	(57) 22.44%
	Uncertain	Incorrect	(197) 77.55%
	False		
6. PIFP is diagnosed via pain scale scores, MRI, and CT in addition to the exclusion of other conditions.			
	True	Correct	(188) 74.01%
	Uncertain	Incorrect	(66) 25.90%
	False		
7. PIFP is hereditary.			
	True	Incorrect	(139) 54.72%
	Uncertain		
	False	Correct	(115) 45.28%
Average of correct answers (%)		51.41%	
Average of incorrect answers (%)		48.35%	

*PIFP: Peripheral Idiopathic Facial Pain.*

**Table 3.** Distribution of participant responses to attitude-related questions on PIFP.

Attitudes towards PIFP				
	Statement	Agree	Uncertain	Disagree
1	PIFP is a serious health issue.	(143) 56.29%	(10) 3.94%	(101) 39.76%
2	My first reaction when experiencing such pain is to consult a clinician.	(89) 35.03%	(114) 44.88%	(51) 20.07%
3	PIFP is prominent in a specific ethnicity.	(67) 26.37%	(103) 40.55%	(84) 33.07%
4	PIFP might interfere with eating, concentrating, speaking and laughing.	(111) 43.70%	(73) 28.74%	(70) 27.56%
5	PIFP is best described as chronic discomfort that is initially localised but may later extend to other areas. It cannot be ascribed to any underlying cause.	(89) 35.03%	(89) 35.03%	(76) 29.92%
6	In my opinion, the best source of information on PIFP is hospital brochures and public health sources.	(52) 20.45%	(78) 30.74%	(124) 48.81%
7	On a scale of 1 to 10, the level of PIFP that can decrease quality of life is above 5 (10 being the most influential).	(100) 36.66%	(99) 31.84%	(55) 21.30%
Average		39.54%	27.00%	33.46%

*PIFP: Peripheral Idiopathic Facial Pain.*

All participants received information on the study prior to commencing the survey. This included information on the study’s aim and objectives. In addition, all participants were informed that involvement is voluntary and that the survey can be withdrawn from at any time. Informed consent was obtained electronically before starting the survey. The study was conducted with adequate concern for the privacy of personal information, and participants were assured of confidentiality and anonymity during data collection, analysis and reporting.

### 2.4 Outcome measures

The extracted outcome measures were documented in a password-protected Microsoft Office® 2023 Excel (Microsoft Corporation, Redmond, WA, USA) spreadsheet and included the following variables: gender, age, ethnicity, profession, educational level, familiarity with PIFP and attitudes towards PIFP. Table 4 and **Supplementary Table 1** summarise the outcome measures.

**Table 4.** Influence of respondents’ characteristics on their level of knowledge and attitudes regarding PIFP.

Demographics		Frequency (%)	Knowledge score (mean ± SD)	*p*-value	Attitude score (mean ± SD)	*p*-value
Gender						
	Male	35.20%	4.10 ± 1.76	0.323	7.46 ± 2.20	0.097
	Female	64.80%	3.85 ± 1.54		7.94 ± 2.14	
Nationality						
	Saudi	95.10%	3.89 ± 1.60	0.684	7.71 ± 2.17	0.081
	Non-Saudi	4.90%	4.08 ± 1.38		8.83 ± 1.95	
Age						
	Below 30 years	39.37%	3.80 ± 1.57	0.349	7.97 ± 2.14	0.160
	30 years or above	60.63%	3.99 ± 1.60		7.59 ± 2.19	
Level of education						
	High school and below	47.25%	3.87 ± 1.45	0.714	7.62 ± 2.12	0.505
	Bachelor’s degree and above	52.75%	3.94 ± 1.73		7.82 ± 2.20	
Occupation sector						
	Healthcare sector	17.32%	4.10 ± 1.76	0.323	7.63 ± 2.37	0.633
	Other	82.68%	3.85 ± 1.54		7.80 ± 2.13	
Marital status						
	Married	44.49%	3.94 ± 1.42	0.731	7.68 ± 1.99	0.589
	Unmarried (including widowed and divorced)	55.51%	3.87 ± 1.71		7.83 ± 2.32	

*SD: Standard deviation.*

### 2.5 Assessment of knowledge and attitudes

Participants’ knowledge levels and attitudes regarding PIFP were calculated independently by summing correct answers to all survey questions. A three-item scale was used to evaluate knowledge; one point was given for the correct response and zero for the incorrect one. Each participant’s knowledge score, represented as mean and standard deviation, must not exceed seven points. The total knowledge score had two levels: high knowledge (>5) and low knowledge (≤5). As a continuous variable, the attitude score was computed by totaling the number of correct responses provided by the respondent to a set of seven questions. An appropriate response (Agree) was assigned one point, signifying a positive attitude, while the responses “Partially agree” and “Disagree” were each worth zero points, signifying a negative attitude. Each participant could achieve a maximum score of seven. To determine the mean attitude score for each respondent, the sum of the attitude scores was divided by seven. A good attitude was indicated by a score of 0.5 or higher, whilst a negative attitude was indicated by a score lower than 0.5.

By adding up the respondent’s right answers to seven questions, the practice score was calculated as a continuous variable. One point was given to participants who chose “Yes”, one point for “Sometimes” and zero points for “No”. Each participant could receive a maximum score of 7. Each respondent’s mean practice score was calculated by dividing their overall practice score by seven. Good knowledge interpretation was defined as a score of 1 or higher, and poor knowledge interpretation was defined as a score of less than 1.

### 2.6 Statistical analysis

A descriptive approach was utilised for data analysis, with results displayed as percentages. The total knowledge and attitude scores of the respondents were calculated independently. Chi-square, mean and standard deviation tests were used to examine associations between categorical sociodemographic variables and knowledge/attitude scores. Spearman’s rank correlation coefficient (*p* < 0.05), odds ratios and confidence were applied to explore the relationship between ordinal and continuous variables. IBM Statistical Package for Social Sciences (SPSS) Statistics version 26 (IBM Corporation, Armonk, NY, USA) was used to analyse the data. A two-sided *p*-value of less than 0.05 was considered statistically significant.

## 3. Results

In total, 262 individuals participated in the online survey. Of these, three were excluded for not completing the survey because they were under the age of 18, and five were excluded for living outside the city of Ha’il, where the study was conducted. The survey’s analysis was divided into four sections: (1) assessment of participants’ demographics; (2) assessment of their knowledge regarding PIFP; (3) assessment of their attitudes towards PIFP; and (4) factors that could have affected participants’ attitudes towards PFIP. This was followed by an in-depth analysis and correlation of the three survey sections to postulate links and draw conclusions.

### 3.1 Assessment of participant demographics

The demographic profile indicated a broad spectrum of ages, with the predominant group being between the ages of 18 and 28 (39.37%, 100 individuals). This was closely followed by individuals aged 29 to 38, accounting for 26.77% (68 individuals); see Table 1.

Participants in this study were mostly female (64.8%, 163 participants) and Saudi citizens (95.1%, 237 participants). Participants varied regarding educational achievement, with a significant 41.73% (106 persons) possessing a Bachelor’s degree. A substantial number of individuals were in the educational sector (21.55%, 55 individuals) and students (27.94%, 71 individuals).

The distribution of income levels was wide-ranging, with 36.23% (92 persons) reporting a monthly income of less than 3000 Saudi Arabian Riyals. The marital status data revealed that 44.49% (113 persons) were married.

### 3.2 Assessment of participants’ knowledge of PIFP

Participants demonstrated a moderate level of knowledge about PIFP, with an average correct response percentage of 51.41%, according to the survey (Tables 2 and 4). Surprisingly, 96.90% (246 respondents) correctly recognised dental difficulties, infections and nerve abnormalities as prevalent factors contributing to PIFP, demonstrating a high degree of understanding of the condition’s origin. However, there were clear misconceptions in other domains. Of all respondents, only 46.06% (117 individuals) properly acknowledged that the female gender experiences a higher degree of PIFP. On the other hand, a considerable percentage of participants (53.93%, 137 individuals) were unsure, indicating a lack of understanding regarding the prevalence of PIFP in relation to gender.

The survey also investigated participants’ impression of age as a factor in PIFP. It found that a significant 69.69% (177 respondents) inaccurately recognised the age range for PIFP as above 70 years, while only 30.31% (77 respondents) correctly answered this question. Questions on the geographical distribution of PIFP revealed additional misunderstandings, as 55.90% (142 respondents) incorrectly believed that PIFP is more frequent in underdeveloped nations. Of all respondents, only 44.90% (112 individuals) accurately disagreed with or expressed uncertainty about this assertion.

Knowledge of the therapy and diagnosis of PIFP varied. Of all respondents, only 22.44% (57 individuals) accurately recognised that antihistamine medication is not effective in treating PIFP. In contrast, the majority (77.55%, 197 respondents) either had doubts or provided wrong answers. On the other hand, a high percentage of respondents (74.01%, 188 individuals) correctly identified the comprehensive diagnostic method for PIFP.

There was confusion regarding the hereditary nature of PIFP, with 54.72% (139 respondents) mistakenly believing PIFP to be hereditary, whereas 45.28% (115 respondents) rightly disagreed.

### 3.3 Assessment of participants’ attitudes towards PIFP

The responses of the 254 participants regarding PIFP exhibited diversity in views, reflecting wider society and personal health concepts (Tables 3 and 4). Of all participants, 56.29% (143 individuals) recognised the seriousness of PIFP.

Nevertheless, the likelihood of immediately seeking medical assistance upon the onset of PIFP symptoms was significantly low: only 35.03% (89 participants) expressed their intention of promptly visiting a healthcare professional. The respondents’ views were further clarified by their preference for information sources regarding PIFP. Approximately half (48.81%, 124 participants) showed doubt regarding hospital brochures and public health sources as the most reliable means of acquiring information.

### 3.4 Factors potentially affecting participants’ attitudes towards PIFP

The survey’s findings revealed a complex link between demographic and personal characteristics upon examining elements that contribute to negative attitudes and disagreements regarding PIFP (**Supplementary Table 1**). Gender played a crucial role, as 30.9% of female participants disagreed with the statement that PIFP is a significant health concern, whereas 45.9% of males disagreed. This discrepancy highlights a disparity in how PIFP’s severity is perceived based on gender, indicating a greater level of concern among women than men (*p*-value: 0.029).

The heterogeneity in opinions was influenced by marital status, although to a lower degree. Married individuals had a slightly lower percentage of disagreement (33.33%) about the seriousness of PIFP compared to those who were unmarried, divorced or widowed (38.41%). However, this difference was not statistically significant (*p*-value: 0.482). Participants’ nationality had a significant impact on their beliefs, as 37.08% of Saudi participants underestimated the significance of PIFP, a feeling that was only shared by 16.67% of non-Saudi participants. The substantial *p*-value of 0.001 indicates that nationality was a major factor influencing the impact of cultural and socioeconomic contexts on health perceptions.

In contrast, age and educational levels had a minimal effect on views towards PIFP. The disagreement rates for individuals below and above 30 years of age were 35.34% and 36.76%, respectively. These results suggest that age does not have a significant influence on disagreement rates, as indicated by a *p*-value of 0.768. Similarly, education level had no significant impact on views, as the disagreement rates were similar among participants with different educational backgrounds (high school or below: 35.21%; Bachelor’s degree or above: 36.46%; *p*-value: 0.968).

The occupational sector, specifically employment in the healthcare profession, did not have a significant impact on perceptions of the significance of PIFP (40.82% of healthcare workers disagreed compared to 34.98% of those in other sectors; *p*-value: 0.550).

## 4. Discussion

Following the analysis of sociodemographic characteristics, knowledge levels and attitudes towards PIFP among residents of Ha’il, Saudi Arabia, this study provides a detailed understanding of public awareness and perceptions of this condition. The results from each segment of the work contribute to a cohesive conclusion that emphasises the urgent requirement for improved public health education and awareness campaigns customised to address the specific needs and misconceptions common within the community.

The evaluation of participants’ knowledge of PIFP revealed a moderate level of general awareness but identified notable deficiencies in the comprehension of certain features of the disorder, such as its aetiology, gender bias and age of onset. This highlights specific areas that require focused efforts for educational enhancement. In comparison, one cross-sectional observational study conducted across multiple cities in Saudi Arabia (Mandorah *et al*. [23], 2024) reported lower awareness of PIFP, with 42.3% of participants demonstrating poor knowledge. In the current study, misinformation regarding the treatment and genetic inheritance of PIFP highlights the necessity for the clear and precise transmission of reliable health information. The statistics indicate a pressing need for focused educational programs to tackle these knowledge gaps and enhance the overall comprehension of PIFP among the public [12, 15]. This aligns with previous research by Mandorah *et al*. [23] (2024) highlighting the need for targeted educational programs, including integrating orofacial pain education into dental curricula, to improve awareness and management of PIFP. Similarly, the British & Irish Society for Oral Medicine has developed patient information leaflets to provide clear and accurate information about PIFP, aiming to correct misconceptions and inform the public [24]. Moreover, the differing degrees of disagreement regarding the severity of the condition among various demographic groups in this study highlight the need for focused educational efforts to reduce these discrepancies and improve the overall comprehension and awareness of PIFP within the community.

The observed reluctance to promptly seek medical advice regarding PIFP underscores significant barriers to health-seeking behaviour and trust in healthcare providers. This aligns with findings from a retrospective study (Brennan *et al*. [25], 2013) emphasising that trust is crucial for patients and may serve as an indicator of their evaluation of healthcare quality. Additionally, the variability in confidence across information sources highlights the necessity for healthcare providers to engage more effectively with the public. The reliance on unconventional health information sources suggests a disconnect between the healthcare system and the community it serves. Addressing this gap presents an opportunity for healthcare providers to build trust and credibility, particularly concerning conditions like PIFP that may be less familiar to the general public.

Furthermore, it is essential to note that the influence of factors such as gender and nationality on attitudes towards PIFP provides an understanding of the social dynamics that determine health beliefs and attitudes in Ha’il. Maarbjerg *et al*. [22] (2017) identified demographic factors influencing the clinical presentation and diagnosis of PIFP, underscoring the importance of culturally sensitive health communication strategies. Therefore, the disparities in recognising the seriousness of PIFP based on gender and the substantial influence of nationality on health attitudes emphasise the significance of implementing culturally sensitive and inclusive health communication techniques.

The findings of this research indicate that a comprehensive strategy is needed to enhance public health interventions for PIFP [26]. First, they highlight the importance of creating specific, culturally sensitive educational programs that target and solve recognised gaps in knowledge and beliefs. Such programs could utilise several communication channels, such as social media, community outreach and healthcare settings, to guarantee a broad and significant influence. Furthermore, it is essential to improve the proficiency of healthcare practitioners to successfully convey information regarding PIFP, including its diagnosis, therapeutic alternatives and management strategies. This encompasses instruction on culturally sensitive patient education and the implementation of patient-centred communication approaches. Furthermore, the results strongly suggest the need for healthcare practitioners, educators and community leaders to work together to create a nurturing atmosphere that promotes prompt and suitable health-seeking behaviours. Building trust and credibility in health information sources is of utmost importance, necessitating the use of consistent, clear and accurate health messaging that aligns with the community’s requirements and preferences.

Studies on PIFP extend far beyond the realm of the medical condition, as PIFP profoundly impacts patients’ lives and requires a comprehensive, multidisciplinary approach. Training for healthcare providers is essential to ensure accurate diagnosis, effective treatment planning and prevention strategies. Multidisciplinary teams should collaborate to create treatment plans that prioritise patients’ autonomy and quality of life while minimising pain and crises. Avoiding unnecessary or iatrogenic procedures is critical, as such interventions may exacerbate patient suffering, delay diagnosis and worsen prognosis. This study underscores the need for targeted public health policies, educational programs and tailored communication strategies to address misconceptions and knowledge gaps that hinder the effective prevention and treatment of PIFP. The findings can also guide interventions by identifying groups with limited knowledge or negative attitudes, ensuring that resources are directed where they are most needed. Advancements in PIFP research hold the potential to refine therapeutic approaches, leading to more personalised and effective treatments for a range of pain conditions.

## 5. Conclusions

This study underscores the urgent need for focused educational initiatives to address knowledge gaps and misconceptions about PIFP. Enhancing public trust in healthcare providers and improving access to reliable health information are essential for progress. By examining the relationship between demographic factors, knowledge and attitudes regarding PIFP in Ha’il, Saudi Arabia, this work provides valuable insights for public health practitioners, policymakers and healthcare professionals. Implementing culturally sensitive health communication strategies and education tailored to the region’s unique demographic and cultural context can significantly improve awareness, attitudes and responses regarding PIFP, ultimately enhancing quality of life and health outcomes.

## 6. Limitations

This study has some limitations, including potential selection bias due to the online survey format and reliance on social media for dissemination, which may have skewed the sample towards younger, more educated individuals with internet access. The use of convenience sampling and self-reported surveys potentially introduced recall bias and limited traceable patient data. Additionally, the cross-sectional design prevented the analysis of variable correlations, and the lack of comparable research on PIFP knowledge and attitudes restricted the ability to contextualise findings. Future cohort studies and interdisciplinary approaches are needed to validate the results and advance pain management practices.

## Supplementary Material

Supplementary material associated with this article can be found, in the online version, at https://files.jofph.com/files/article/1933043990940008448/attachment/Supplementary%20material.docx.


