# Supplementary material for: Perceptions of persistent idiopathic facial pain: a comprehensive study of adults in Ha’il city, Saudi Arabia
Source: J Oral Facial Pain Headache. 2025 Jun 12;39(2):146–54. doi: 10.22514/jofph.2025.033 (PMC12520420; doi:10.22514/jofph.2025.033)
Supplement: Supplementary file 1 [file Supplementary-material.docx]

Supplementary material

Supplementary Table 1. Factors affecting the good attitude of participants towards PIFP.

| Disagreeing that (PIFP) is a serious health issue | | | *p*-value | OR (CI) |
| --- | --- | --- | --- | --- |
| Gender (female *vs.* male) | (Female)  30.9% | (Male)  45.9% | 0.029 | 0.53  (0.37, 0.76) |
| Marital status (married *vs.* others) | (Married)  33.33% | (Other)  38.41% | 0.482 | 0.80  (0.48, 1.35) |
| Nationality (Saudi *vs.* non-Saudi) | (Saudi)  37.08% | (Non-Saudi)  16.67% | 0.001 | 2.97  (1.96, 4.51) |
| Age (below 30 *vs.* 30 and above) | (Below 30)  35.34% | (Above 30)  36.76% | 0.768 | 0.93  (0.65, 1.34) |
| Level of education (high school degree and below *vs.* Bachelor’s degree or above) | (High school and below)  35.21% | (Bachelor’s degree or above)  36.46% | 0.968 | 1.06  (0.60, 1.87) |
| Occupational sector (healthcare *vs.* other) | (Healthcare)  40.82% | (Other)  34.98% | 0.550 | 1.28  (0.68, 2.43) |
| Disagreeing that the first reaction when experiencing such pain is to consult a clinician | | | *p*-value | OR (CI) |
| Gender (female *vs.* male) | (Female)  15.8% | (Male)  18.8% | 0.576 | 1.28  (0.76, 2.15) |
| Marital status (married *vs.* others) | (Married)  14.04% | (Other)  18.84% | 0.340 | 0.65  (0.38, 1.10) |
| Nationality (Saudi *vs.* non-Saudi) | (Saudi)  17.08% | (Non-Saudi)  8.33% | 0.054 | 2.58  (1.42, 4.69) |
| Age (below 30 *vs.* 30 and above) | (Below 30)  17.24% | (Above 30)  16.18% | 0.848 | 1.07  (0.64, 1.81) |
| Level of education (high school degree and below *vs.* Bachelor’s degree or above) | (High school and below)  26.76% | (Bachelor’s degree or above)  12.71% | 0.012 | 0.40  (0.20, 0.79) |
| Occupational sector (healthcare *vs.* other) | (Healthcare)  12.24% | (Other)  17.73% | 0.477 | 0.65  (0.26, 1.64) |
| Disagreeing that PIFP is prominent in a specific ethnicity | | | *p*-value | OR (CI) |
| Gender (female *vs.* male) | (Female)  32.7% | (Male)  34.1% | 0.961 | 0.932  (0.644, 1.347) |
| Marital status (married *vs.* others) | (Married)  29.82% | (Other)  36.96% | 0.290 | 0.73  (0.43, 1.23) |
| Nationality (Saudi *vs.* non-Saudi) | (Saudi)  33.75% | (Non-Saudi)  33.33% | 0.880 | 1.018  (0.704, 1.471) |
| Age (below 30 *vs.* 30 and above) | (Below 30)  35.34% | (Above 30)  29.41% | 0.363 | 1.089  (0.683, 1.735) |
| Level of education (high school degree and below *vs.* Bachelor’s degree or above) | (High school and below)  33.80% | (Bachelor’s degree or above)  33.70% | 1.000 | 0.99  (0.56, 1.78) |
| Occupational sector (healthcare *vs.* other) | (Healthcare)  38.78% | (Other)  32.51% | 0.507 | 1.31  (0.69, 2.51) |
| Disagreeing that PIFP might interfere with eating, concentrating, speaking and laughing | | | *p*-value | OR (CI) |
| Gender (female *vs.* male) | (Female)  57.6% | (Male)  61.2% | 0.666 | 0.88  (0.53, 1.45) |
| Marital status (married *vs.* others) | (Married)  60.53% | (Other)  57.25% | 0.691 | 1.15  (0.69, 1.90) |
| Nationality (Saudi *vs.* non-Saudi) | (Saudi)  58.75% | (Non-Saudi)  41.25% | 0.008 | 2.01  (1.22, 3.32) |
| Age (below 30 *vs.* 30 and above) | (Below 30)  57.76% | (Above 30)  59.56% | 0.773 | 0.94  (0.57, 1.54) |
| Level of education (high school degree and below *vs.* Bachelor’s degree or above) | (High school and below)  61.97% | (Bachelor’s degree or above)  57.46% | 0.608 | 0.83  (0.47, 1.45) |
| Occupational sector (healthcare *vs.* other) | (Healthcare)  48.98% | (Other)  61.08% | 0.167 | 0.61  (0.33, 1.15) |
| Disagreeing that PIFP is best described as chronic discomfort that is initially localised but may later extend to other areas. It cannot be ascribed to any underlying cause. | | | *p*-value | OR (CI) |
| Gender (female *vs.* male) | (Female)  4.2% | (Male)  1.00% | 0.174 | 5.16  (0.59, 44.84) |
| Marital status (married *vs.* others) | (Married)  5.80% | (Other)  94.20% | 0.024 | 0.004  (0.0014, 0.0112) |
| Nationality (Saudi *vs.* non-Saudi) | (Saudi)  3.33% | (Non-Saudi)  1.00% | 0.312 | 4.10  (0.45, 37.18) |
| Age (below 30 *vs.* 30 and above) | (Below 30)  1.86% | (Above 30)  5.15% | 0.248 | 0.32  0.06 to 1.63 |
| Level of education (high school degree and below *vs.* Bachelor’s degree or above) | (High school and below)  1.41% | (Bachelor’s degree or above)  3.87% | 0.547 | 2.82  (0.34, 23.31) |
| Occupational sector (healthcare *vs.* other) | (Healthcare)  10.20% | (Other)  1.48% | 0.0075 | 7.58  (1.75, 32.89) |
| Disagreeing that the best source of information on PIFP is hospital brochures and public health sources | | | *p*-value | OR (CI) |
| Gender (female *vs.* male) | (Female)  25.5% | (Male)  20.0% | 0.313 | 1.37  (0.76, 2.49) |
| Marital status (married *vs.* others) | (Married)  26.32% | (Other)  21.74% | 0.484 | 1.29  (0.72, 2.30) |
| Nationality (Saudi *vs.* non-Saudi) | (Saudi)  24.17% | (Non-Saudi)  16.67% | 0.220 | 1.56  (0.84, 2.91) |
| Age (below 30 *vs.* 30 and above) | (Below 30)  17.24% | (Above 30)  26.01% | 0.121 | 0.56  (0.31, 1.04) |
| Level of education (high school degree and below *vs.* Bachelor’s degree or above) | (High school and below)  19.72% | (Bachelor’s degree or above)  25.41% | 0.429 | 1.39  (0.71, 2.72) |
| Occupational sector (healthcare *vs.* other) | (Healthcare)  30.61% | (Other)  22.17% | 0.290 | 1.55  (0.78, 3.09) |
| Disagreeing that on a scale of 1 to 10, the level of PIFP that can decrease quality of life is above 5 (10 being the most influential) | | | *p*-value | OR (CI) |
| Gender (female *vs.* male) | (Female)  21.2% | (Male)  28.2% | 0.267 | 0.68  (0.38, 1.21) |
| Marital status (married *vs.* others) | (Married)  30.70% | (Other)  18.12% | 0.029 | 1.93  (1.07, 3.48) |
| Nationality (Saudi *vs.* non-Saudi) | (Saudi)  24.17% | (Non-Saudi)  16.67% | 0.220 | 1.56  (0.84, 2.91) |
| Age (below 30 *vs.* 30 and above) | (Below 30)  25.86% | (Above 30)  22.06% | 0.507 | 1.19  (0.67, 2.13) |
| Level of education (high school degree and below *vs.* Bachelor’s degree or above) | (High school and below)  29.58% | (Bachelor’s degree or above)  21.55% | 0.237 | 0.65  (0.35, 1.22) |
| Occupational sector (healthcare *vs.* other) | (Healthcare)  26.53% | (Other)  23.15% | 0.755 | 1.20  (0.59, 2.45) |

PIFP: Peripheral idiopathic Facial Pain; OR: Odd Ratio; CI: Confidence Interval.
